# Brain cytoprotection in acute ischemic stroke in the reperfusion era: neuroimmune networks, clinical evidence, and translational barriers

**DOI:** 10.3389/fimmu.2026.1984227

**Published:** 2026-10-21

**Authors:** Xue Gao, Shanshan Tong, Xiaonan Wang, Xuan Zhou, Qiong Chen, Jinxiang Wang, Fan Wu

**Affiliations:** 1 Department of Neurology, Anhui No.2 Provincial People’s Hospital, Hefei, Anhui, China; 2 Faculty of Chinese Medicine, Macau University of Science and Technology, Taipa, Macao SAR, China; 3 Quality Control Office, Anhui No.2 Provincial People’s Hospital, Hefei, Anhui, China; 4 Research and Education Office, Guilin Municipal Hospital of Traditional Chinese Medicine, Guilin, Guangxi Zhuang Autonomous Region, China; 5 Department of Medical Laboratory Center, Anhui No.2 Provincial People’s Hospital, Hefei, Anhui, China; 6 Department of Urology, Pelvic Floor Disorders Center, The Seventh Affiliated Hospital, Sun Yat-sen University, Shenzhen, China; 7 Department of Otorhinolaryngology, Head & Neck Surgery, The First Affiliated Hospital of Anhui Medical University, Hefei, China

**Keywords:** acute ischemic stroke, brain cytoprotection, clinical translation, ischemic penumbra, mechanical thrombectomy, neuroprotection, neurovascular unit, no-reflow

## Abstract

Intravenous thrombolysis and mechanical thrombectomy have transformed acute ischemic stroke (AIS) care, yet angiographic recanalization does not guarantee adequate tissue reperfusion or long-term functional independence. Brain cytoprotection has therefore expanded from neuron-centered rescue to preservation of the ischemic penumbra, neurovascular unit, blood-brain barrier (BBB), and microcirculation. Using a systems-immunology perspective, this clinically oriented narrative review examines ischemia-reperfusion injury as a dynamic network linking danger-associated molecular pattern sensing, microglia and astrocytes, circulating leukocytes, platelets and complement, endothelium and pericytes, cytokine signaling, edema, no-reflow, and regulated cell-death programs. We integrate classic and recent evidence for excitotoxic, redox and mitochondrial, inflammatory, barrier, microvascular, and cell-death targets; evaluate representative pharmacologic and nonpharmacologic interventions; and distinguish negative primary endpoints from exploratory subgroup or surrogate signals. Confirmatory excitotoxicity trials have remained negative overall. Selected multitarget agents have shown benefit in specific Chinese populations, whereas immune-, edema-, and no-reflow-directed approaches have yielded stage-specific but inconsistent safety, imaging, and functional results. Statins, nitric oxide donors, and phosphodiesterase inhibition have plausible vascular or immunomodulatory actions, but available trials do not establish them as acute cytoprotective therapies. Future development should align the right mechanism, tissue phenotype, treatment time, exposure, target-engagement measure, and patient-centered endpoint, followed by multicenter preclinical testing and independent cross-regional clinical replication. This end-to-end framework reframes brain cytoprotection as precision modulation of a spatially and temporally evolving neuroimmune-vascular system rather than a single undifferentiated treatment class.

## Graphical Abstract

**Figure:**
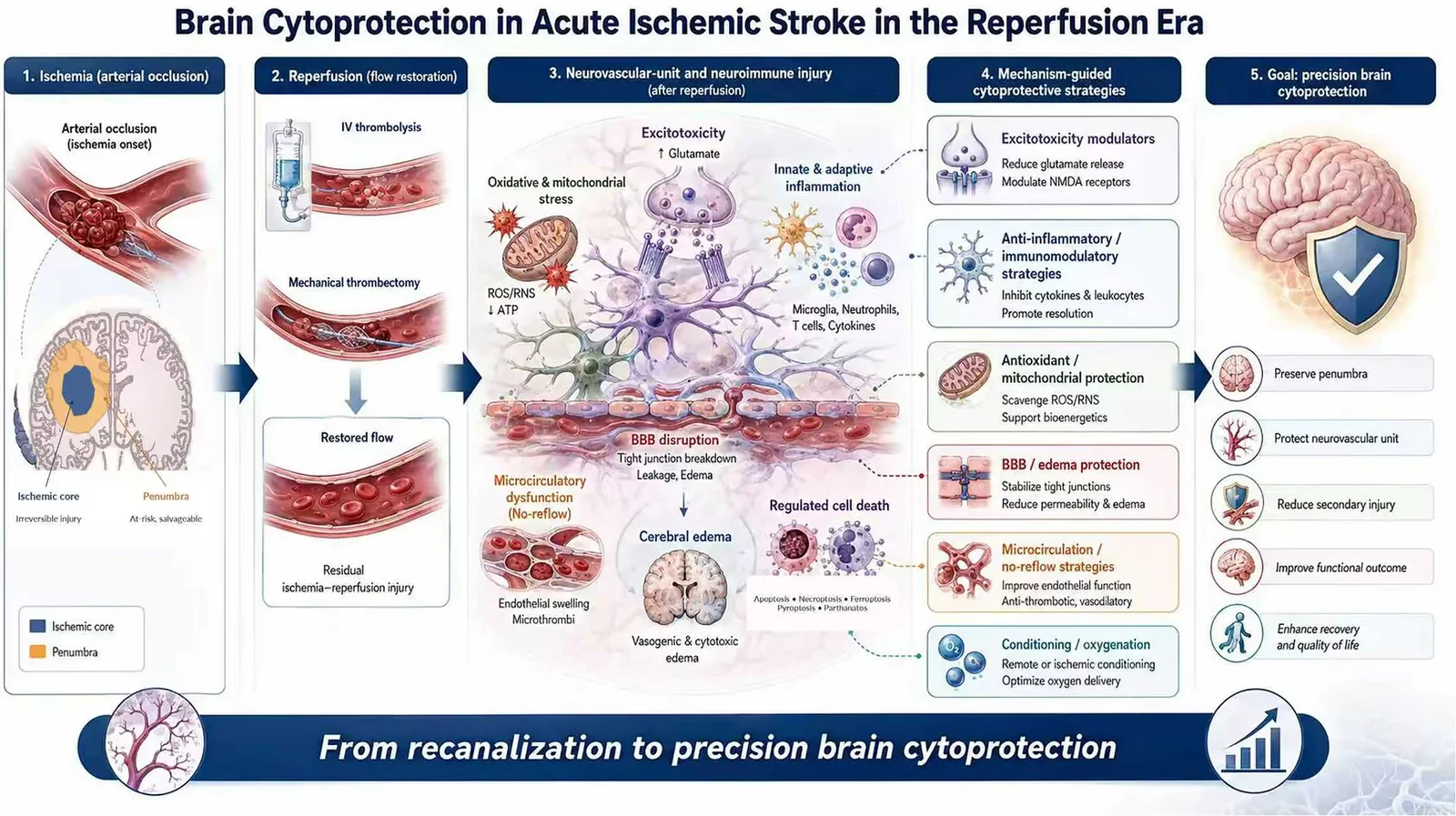
From recanalization to precision brain cytoprotection in acute ischemic stroke. The ischemic core is depicted as a localized region surrounded by penumbra in the same hemisphere, and mechanical thrombectomy is shown as an intraluminal clot-retrieval procedure. Reperfusion restores large-vessel flow but may leave residual neurovascular, neuroimmune, microcirculatory, edematous, and regulated cell-death injury. Mechanism-guided cytoprotection aims to preserve salvageable tissue and the neurovascular unit by matching therapeutic targets to tissue state, timing, and reperfusion context, ultimately improving meaningful functional recovery.

## 1 Introduction: why brain cytoprotection is still needed after successful reperfusion

### 1.1 Transformative advances in reperfusion therapy and the residual treatment gap

#### 1.1.1 From restoring arterial patency to improving functional outcomes

The immediate cause of acute ischemic stroke (AIS) is focal hypoperfusion due to cerebral arterial occlusion. By restoring blood flow, intravenous thrombolysis (IVT) and mechanical thrombectomy have transformed a clinical landscape that was previously dominated by supportive care. The HERMES collaboration pooled individual-patient data from 1, 287 participants in five early randomized thrombectomy trials and showed that mechanical thrombectomy significantly shifted the 90-day modified Rankin Scale (mRS) distribution toward less disability, with an adjusted common odds ratio (cOR) of 2.49 and a number needed to treat of approximately 2.6 for improvement of at least one mRS category (1). DAWN and DEFUSE 3 further demonstrated that thrombectomy benefit could be extended to 16–24 h after onset in patients selected by a clinical-core mismatch or perfusion imaging showing salvageable tissue (2, 3). These studies established reperfusion as the cornerstone of acute stroke treatment and shifted the concept of the “time window” from clock time alone to tissue time determined jointly by infarct core, penumbra, and collateral circulation.

The success of reperfusion therapy, however, has not eliminated the burden of disability. Even when large-vessel recanalization is technically successful, a substantial proportion of patients do not regain functional independence. Explanations include an already large irreversible core before recanalization, inadequate distal microvascular perfusion, cerebral edema, blood-brain barrier (BBB) disruption, hemorrhagic transformation, systemic complications, and damage to strategically important brain regions or functional networks. The 2026 American Heart Association/American Stroke Association guideline continues to prioritize rapid thrombolysis, endovascular therapy, and organized stroke-unit care, while not recommending pharmacologic or nonpharmacologic neuroprotection as an undifferentiated routine treatment category for improving functional outcomes (4). The central question is therefore not whether brain protection is biologically conceivable, but whether a given intervention can provide a sufficiently robust incremental benefit in the right patient, tissue state, and treatment workflow.

#### 1.1.2 Large-vessel recanalization is not synonymous with tissue reperfusion

Three levels should be distinguished: angiographic recanalization, tissue-level reperfusion, and patient-level functional recovery. Successful large-vessel recanalization is necessary for tissue salvage in many patients, but it is not sufficient. Animal studies have shown that after reopening of the middle cerebral artery, pericytes may remain contracted because of oxidative-nitrative stress, with red blood cell trapping and impaired capillary reflow (5). More recent human imaging studies also indicate that tissue-level hypoperfusion can persist after successful thrombectomy. In a *post hoc* pooled analysis of thrombectomy studies, Rivet et al. observed persistent tissue hypoperfusion in approximately 30% of patients, which was associated with attenuation of the clinical benefit of successful recanalization (6). In a prospective single-center cohort of 65 patients, Horie et al. used postprocedural perfusion imaging and found that residual hypoperfusion, defined by several thresholds, was associated with infarct expansion and unfavorable outcomes (7). These clinical data are observational and do not establish that no-reflow necessarily causes poor outcome, but they move microcirculatory protection from an animal hypothesis toward a measurable human phenotype.

Accordingly, “futile reperfusion” and “no-reflow” should not be used interchangeably. Futile reperfusion generally describes failure to achieve a favorable functional outcome despite successful large-vessel recanalization and is a composite phenomenon influenced by core size, infarct location, age, comorbidities, speed of recanalization, and complications. No-reflow more specifically refers to inadequate restoration of microcirculatory or tissue perfusion. The potential value of brain cytoprotection lies in addressing tissue injury that persists beyond restoration of arterial patency without compromising reperfusion therapy.

### 1.2 From neuroprotection to brain cytoprotection

#### 1.2.1 Conceptual evolution and implications for research

Traditional neuroprotection has often treated neuronal death as the principal endpoint, whereas ischemic injury simultaneously affects astrocytes, oligodendrocytes, cerebral microvascular endothelial cells, pericytes, the basement membrane, and the extracellular matrix. STAIR XI therefore advocated the terms brain cytoprotection or cerebroprotection to emphasize preservation of the entire neurovascular unit rather than neurons alone (8). Fisher and Savitz further argued that, in the reperfusion era, brain cytoprotection must consider whether an intervention reaches ischemic tissue, remains compatible with thrombolysis or thrombectomy, and produces imaging effects that translate into long-term functional benefit (9). The latest STAIR update identifies explicit definition of the intended biological target, development of mechanism-relevant biomarkers, and selection of clinically meaningful outcomes as priorities for trial design (10).

This conceptual shift expands the object of brain protection from survival of a single cell type to a multilevel therapeutic problem: preserving salvageable tissue in the hyperacute phase; limiting oxidative, inflammatory, and barrier injury during reperfusion; and sustaining microcirculation, reducing edema, and protecting neural networks that retain potential for functional recovery after recanalization. It also means that no intervention should be considered clinically cerebroprotective solely because it reduces infarct volume in an animal model.

#### 1.2.2 Scope and core questions of this review

This review focuses on adult AIS and operationally considers interventions initiated within 72 h of symptom onset or last known well. The 72-h boundary is not an accepted biological cutoff; it allows us to encompass hyperacute penumbral preservation, peri-reperfusion injury, and early secondary inflammation or edema. Reperfusion therapy, cytoprotection, and neural repair overlap temporally, but interventions begun weeks after stroke—such as cell therapy, exosomes, brain-computer interfaces, and rehabilitation—are outside the systematic scope of this article.

A systems-immunology lens is used because immune signaling in AIS cannot be separated from vascular and tissue context. Danger sensing, microglial and astrocytic responses, cytokines, complement, platelets, leukocyte trafficking, endothelial activation, BBB permeability, and no-reflow are treated as a coupled network whose net effect changes across compartments and time. This framing is directly relevant to immunology while retaining clinical endpoints as the standard for therapeutic claims.

A recent review by Boltze and Fisher comprehensively framed cytoprotection in the recanalization era around pathophysiology, improved preclinical models, imaging, biomarkers, and trial design (11). The present review is complementary and differs in four deliberate respects: it centers neuroimmune-thromboinflammatory and BBB/no-reflow crosstalk; incorporates major 2025–2026 trials; separates primary outcomes from surrogate or subgroup signals; and links target engagement, patient-centered outcomes, and independent replication in a single translational roadmap.

Within this scope, we address four interrelated questions. First, which components of ischemia-reperfusion injury constitute biologically plausible and measurable therapeutic targets? Second, why have so many classic candidate agents failed in clinical trials? Third, what do recent positive or borderline-positive trials actually support—and what do they not support? Fourth, how should next-generation trials prospectively align mechanism, tissue time, drug exposure, patient selection, and functional outcomes? Rather than cataloging agents chronologically, the review organizes evidence along the sequence of biological target, representative intervention, clinical validation, and translational interpretation.

**Figure 1** provides an overview of the major pathophysiological cascades and their spatiotemporal therapeutic windows. **Table 1** complements the figure by comparing the potential clinical role and evidence maturity of each biological domain, including neurovascular, immune, microcirculatory, and regulated cell-death targets.

**Figure 1:**
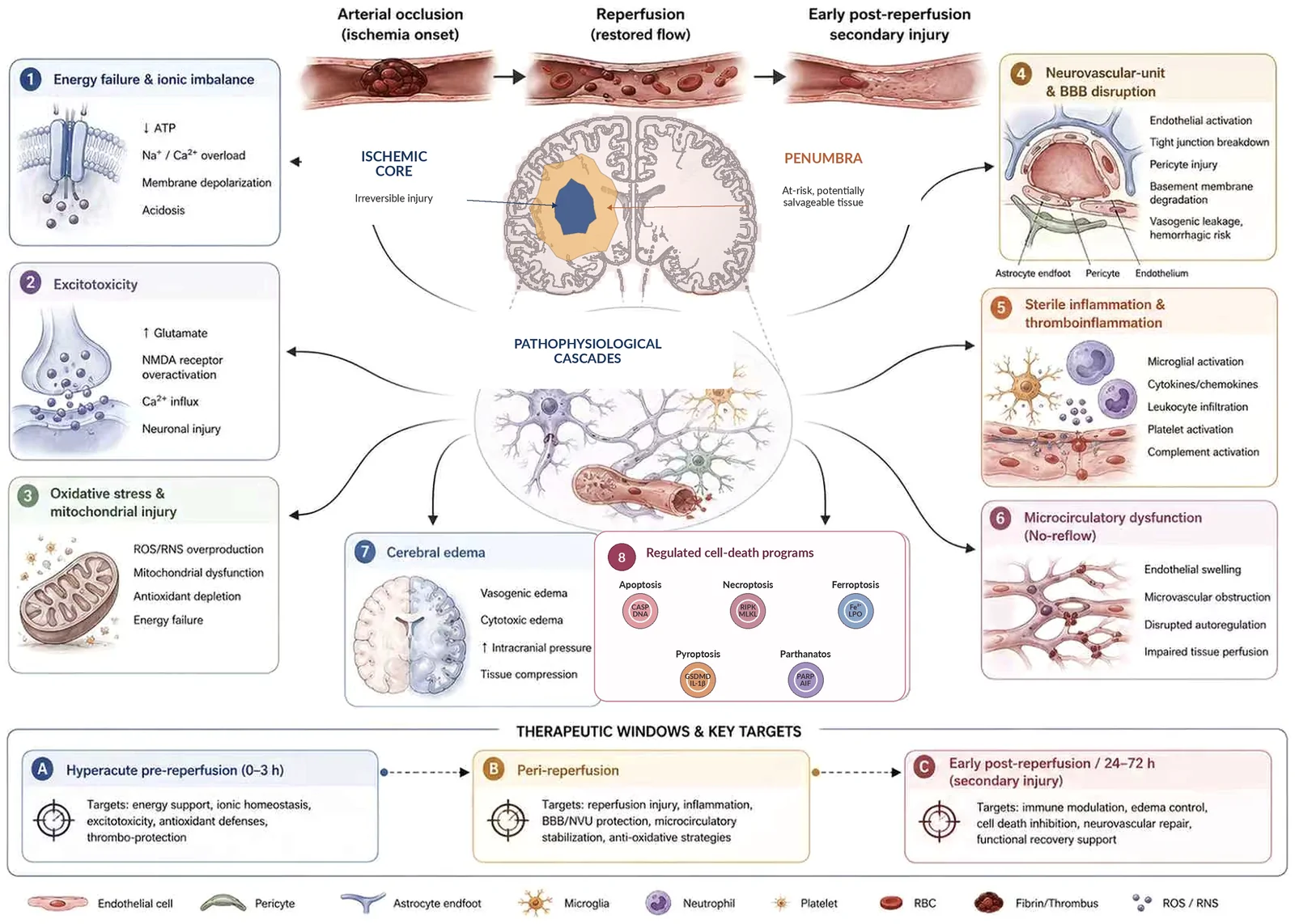
Pathophysiological cascades and spatiotemporal targets of brain cytoprotection in acute ischemic stroke. The ischemic core is shown as a localized region of irreversible injury surrounded within the same vascular territory by at-risk, potentially salvageable penumbra. Arterial occlusion initiates energy failure and ionic imbalance, followed by excitotoxicity and oxidative and mitochondrial injury. Although reperfusion is essential for tissue rescue, it may coexist with or amplify neurovascular-unit dysfunction, BBB disruption, sterile inflammation and thromboinflammation, no-reflow, cerebral edema, and overlapping regulated cell-death programs, including apoptosis, necroptosis, ferroptosis, pyroptosis, and parthanatos. The five programs are represented by non-anatomical pathway symbols and are not intended to imply mutually exclusive morphologic cell types. The lower panel depicts hyperacute pre-reperfusion, peri-reperfusion, and early post-reperfusion/24–72-h therapeutic windows. RBC, red blood cell; ROS, reactive oxygen species; RNS, reactive nitrogen species.

**Table 1:** Major biological domains of ischemia-reperfusion injury, potential clinical roles, and maturity of evidence.

Biological domain	Principal targets/phenotypes	Potential clinical role	Current evidence maturity	Representative evidence
Ischemic core and penumbra	Salvageable tissue, core growth, collateral circulation	Bridging before reperfusion; selecting patients who still have a viable tissue target	Imaging selection is established for reperfusion RCTs; prediction of drug response is not established	(1–3, 12–14)
Excitotoxicity	Downstream NMDA receptor–PSD-95 signaling	Hyperacute reduction of synaptic toxic cascades	Strong preclinical evidence; multiple confirmatory clinical trials have been negative overall	(16–18, 43, 45–47)
Oxidative and mitochondrial injury	NOX4, free radicals, mitochondrial bioenergetics, and intercellular metabolic support	Limit oxidative injury and maintain energy before and after reperfusion	Predominantly animal mechanistic evidence; mitochondrial transfer remains experimental; clinical mediation by redox or mitochondrial pathways is unproven	(5, 19, 20)
Inflammation and the blood-brain barrier	TLR4, IL-1/IL-6, leukocyte trafficking, endothelial barrier	Modulation of peri-reperfusion and later secondary injury	Results are inconsistent from early clinical development through phase III; target engagement often dissociates from functional benefit	(15, 21–27, 44, 55)
Cerebral edema	SUR1-TRPM4-related channels; barrier permeability	Reduce mass effect and secondary injury	CHARM was negative for its primary functional endpoint and was terminated early	(15)
Microcirculatory no-reflow	Pericytes, endothelium, microthrombi, and tissue hypoperfusion	Rescue tissue perfusion after successful recanalization	Animal mechanisms and human observational phenotypes are available; targeted RCTs are lacking	(5–7)
Regulated cell death	Apoptosis, necroptosis, ferroptosis, pyroptosis, and parthanatos	Delay ongoing cellular loss and interrupt inflammatory amplification	Predominantly cellular and animal evidence; programs overlap and clinically specific target-engagement biomarkers are lacking	(28–32)

## 2 Biological basis and spatiotemporal targets of brain cytoprotection

### 2.1 The ischemic penumbra: from fixed time windows to tissue time

#### 2.1.1 The core and penumbra are not static compartments

The concept of the ischemic penumbra proposed by Astrup, Siesjö, and Symon recognizes distinct functional thresholds of cerebral blood flow: when perfusion falls below the threshold required to sustain neuronal electrical activity but remains above the threshold for loss of membrane integrity, tissue function is suppressed yet the tissue may remain viable (12). This concept provides the theoretical foundation for brain protection, but modern imaging shows that the boundary between core and penumbra is not fixed. The severity and duration of flow reduction, collateral circulation, gray-white matter differences, and metabolic demand all influence tissue fate (13, 14). Population-level estimates such as “neurons lost per minute” therefore cannot substitute for individual tissue status.

DAWN and DEFUSE 3 selected patients using imaging or clinical-core mismatch and demonstrated that some slow progressors retain salvageable tissue beyond conventional time windows (2, 3). These trials directly support late-window reperfusion, not late-window pharmacologic cytoprotection. Their major methodological implication is nevertheless crucial: a brain-protection trial that does not measure whether salvageable tissue is still present at treatment may mix patients who no longer have a viable therapeutic target with those who remain biologically capable of responding.

#### 2.1.2 Imaging-defined penumbra is a biological approximation, not an absolute ground truth

Computed tomography perfusion and magnetic resonance perfusion-diffusion mismatch are among the most practical clinical tools for tissue phenotyping, but any single threshold is only an approximation of a complex physiological state. Leigh et al. emphasized that perfusion thresholds vary with baseline blood flow, time, and gray-white matter differences; defining penumbra solely by hypoperfusion may therefore include benign oligemia or miss metabolically compromised tissue (13). Future cytoprotection trials should combine core volume, perfusion mismatch, collateral status, and recanalization outcome rather than treating a single imaging threshold as an established biomarker of treatment response.

### 2.2 The neurovascular unit and the blood-brain barrier

#### 2.2.1 Multicellular coupling determines tissue fate

Brain metabolism and perfusion depend on dynamic coupling among neurons, astrocytes, oligodendrocytes, cerebral microvascular endothelial cells, pericytes, and immune cells. Excitotoxic neuronal injury increases metabolic demand and ionic load; astrocytes contribute to glutamate clearance, ion buffering, and metabolic support; endothelial cells and pericytes regulate capillary permeability and flow; and oligodendrocytes together with axons determine white-matter conduction. Protection of one compartment may therefore fail if another has sustained irreversible injury. This coupled neurovascular-unit framework is summarized with the other major biological domains in **Table 1** (8, 9).

#### 2.2.2 Barrier disruption, cerebral edema, and functional outcome

BBB disruption can promote vasogenic edema, inflammatory-cell entry, and hemorrhagic transformation. In large hemispheric infarction, edema itself can produce mass effect and secondary injury. CHARM tested SUR1-TRPM4-directed intravenous glibenclamide but did not improve the 90-day mRS distribution and increased hypoglycemia; trial-level details and interpretive limitations are discussed in section 4.4.4 (15).

### 2.3 Excitotoxicity: biological plausibility and time dependence

#### 2.3.1 Uncoupling downstream NMDA receptor signaling

Ischemia causes ATP depletion, ion-pump failure, membrane depolarization, and glutamate release, followed by excessive N-methyl-D-aspartate (NMDA) receptor activation and engagement of multiple downstream injury pathways. Broad blockade of NMDA receptors can disrupt normal synaptic function. Aarts et al. therefore targeted the interaction between NMDA receptors and the PSD-95 scaffold protein and observed reduced injury in cultured neurons and a rat model of focal ischemia (16). The attraction of this strategy is that it seeks to preserve physiological receptor function while interrupting a pathological signaling complex, but its therapeutic window depends on drug delivery to the synapse before irreversible injury has occurred.

ENACT evaluated NA-1 in small iatrogenic ischemic lesions caused by endovascular aneurysm repair and identified favorable signals in the number or volume of diffusion-weighted imaging lesions (17). This setting has predictable timing of ischemic risk, relatively small lesions, and well-defined drug administration, and is therefore not equivalent to spontaneous large-vessel occlusion. ENACT can be viewed as proof of concept that the target is testable in a human cerebral ischemic setting, but it cannot directly predict functional benefit in major spontaneous stroke.

#### 2.3.2 Interactions between cytoprotective drugs and the reperfusion workflow

The nerinetide program illustrates that standard reperfusion therapy can alter the exposure or effect of a candidate cytoprotective agent. An alteplase-related pharmacokinetic interaction was proposed after ESCAPE-NA1, but this hypothesis required prospective testing and did not establish clinical efficacy. The mechanistic and confirmatory evidence is considered once in detail in sections 3.3.2 and 4.1; more broadly, every cytoprotection program should prospectively test compatibility with thrombolysis, thrombectomy, anesthesia, and the intended administration sequence (18).

### 2.4 Oxidative-nitrative stress and mitochondrial networks

#### 2.4.1 Reperfusion can restore energy while amplifying injury

Restored blood flow supplies oxygen and glucose and is essential for cell survival, yet electron-transport-chain dysfunction, calcium overload, and enzymatic generation of free radicals may amplify oxidative injury during early reperfusion. In mouse models, Kleinschnitz et al. identified NOX4 as an important source of post-ischemic oxidative stress, with genetic deletion or inhibition of NOX4 reducing oxidative injury and neurodegeneration (19). Yemisci et al. linked oxidative-nitrative stress to pericyte contraction and impaired microcirculatory reflow (5). Together, these studies show that oxidative stress is not confined to neurons; it can also affect the vasculature and tissue perfusion.

These preclinical findings provide biological support for free-radical scavenging and multitarget drugs, but differences in clinical functional outcomes cannot be used retrospectively to prove that a particular redox pathway mediated benefit. Nelonemdaz, butylphthalide, and edaravone dexborneol have all been attributed antioxidant or mitochondrial-protective properties, yet their clinical studies differ substantially in population, treatment window, and comparator. What must be demonstrated is whether an intervention alters prespecified redox biomarkers in patients and whether those changes mediate tissue preservation and functional benefit.

#### 2.4.2 Mitochondrial injury and intercellular compensation

Mitochondrial dysfunction affects ATP generation, membrane potential, calcium homeostasis, redox balance, and cell-death signaling. Astrocyte-to-neuron mitochondrial transfer is an intriguing experimental example of intercellular metabolic support, but it currently lacks validated human assays, patient-selection markers, or a clinically testable intervention. We therefore treat it as supporting biology rather than as a near-term therapeutic platform (20).

### 2.5 Sterile inflammation, thromboinflammation, and immune modulation

#### 2.5.1 Inflammation is a time- and cell-type-dependent network

Ischemic tissue releases danger-associated signals that activate the endothelium, resident glia, platelets, complement, and peripheral immune cells, promoting adhesion, thromboinflammation, barrier permeability, edema, and microvascular obstruction. These elements form a coupled, time-dependent system: the same cell population or mediator may be injurious, neutral, or reparative according to compartment and phase. Consequently, an actionable immunologic hypothesis must specify the time window, cellular compartment, pathway state, and measurable target-engagement marker rather than assume that broader immunosuppression is preferable.

#### 2.5.2 Multiple points of failure between target engagement and functional benefit

TLR4-binding DNA aptamers showed receptor antagonism and tissue-protective signals *in vitro* and in murine stroke models (21), providing the basis for early clinical development of ApTOLL (22). In SCIL-STROKE, an interleukin-1 receptor antagonist primarily reduced peripheral inflammatory responses; the regimen was safe but did not establish 90-day functional benefit (23). Natalizumab, which targets leukocyte trafficking, did not reduce infarct growth in ACTION, and early functional signals were not replicated in ACTION II; ACTION II even showed a lower probability of achieving excellent outcomes than placebo (24, 25). These results illustrate that changing a peripheral immune marker, reducing migration of a particular cell population, or improving a short-term imaging measure does not necessarily improve long-term function.

Small fingolimod studies illustrate another possible route. In a pilot randomized study of 47 patients treated within 4.5 h, fingolimod plus alteplase was associated with smaller lesions, less hemorrhage, and more favorable 90-day outcomes (26). A separate open-label study with blinded endpoint assessment, including 23 patients per group treated in the 4.5–6 h window, reported perfusion- and collateral-related signals (27). These studies were extremely small, treatment was open label, and multiple endpoints were assessed; they therefore support further testing rather than establishing the efficacy of lymphocyte-redistribution therapy.

The neuroimmune crosstalk linking innate immune sensing, resident and peripheral immune-cell responses, endothelial and platelet activation, BBB injury, and representative immunomodulatory interventions is summarized in **Figure 2**. **Table 1** places this immune-vascular network alongside the other biological domains and makes explicit that target engagement and functional efficacy represent different evidentiary levels.

**Figure 2:**
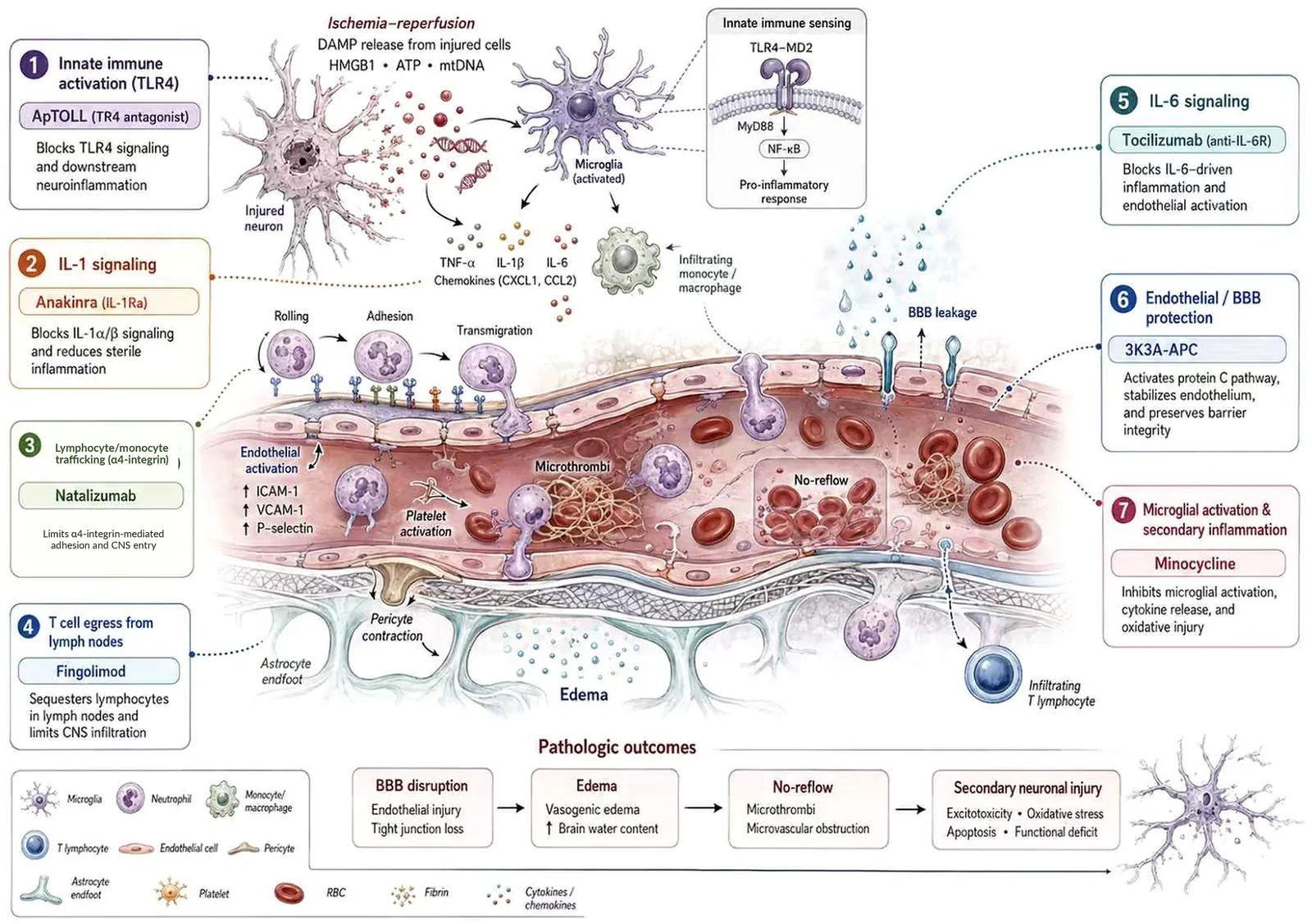
Neuroimmune crosstalk and actionable immunologic targets for brain cytoprotection in acute ischemic stroke. Ischemia-reperfusion activates danger-associated signaling, resident microglia, vascular endothelium, and peripheral immune cells, promoting cytokine and chemokine release, leukocyte recruitment, platelet activation, microthrombosis, BBB disruption, edema, and microcirculatory impairment. Representative interventions discussed in this review include TLR4 antagonism with ApTOLL, IL-1 receptor antagonism, limitation of α4-integrin-mediated lymphocyte and monocyte trafficking with natalizumab, lymphocyte redistribution with fingolimod, IL-6 receptor inhibition with tocilizumab, endothelial and barrier-directed cytoprotection with 3K3A-APC, and minocycline. The lower row summarizes interacting pathologic outcomes rather than a strictly linear causal sequence. These interventions are shown as mechanistically relevant investigational strategies and should not be interpreted as established standard-of-care therapies.

### 2.6 Microcirculatory no-reflow: from experimental mechanism to human phenotype

#### 2.6.1 No-reflow may represent a convergence point for residual injury after recanalization

Microcirculatory no-reflow may involve pericyte contraction, endothelial swelling, microthrombi, leukocyte adhesion, altered rheology, and tissue edema. Animal mechanistic evidence links pericytes and oxidative-nitrative stress to impaired capillary reflow (5), whereas human studies indicate that tissue hypoperfusion may remain measurable after successful thrombectomy and is associated with infarct expansion or unfavorable outcome (6, 7). However, studies use different Tmax, cerebral blood flow, and cerebral blood volume thresholds, producing markedly different no-reflow estimates and indicating that the phenotype has not yet been clinically standardized.

Human evidence is currently dominated by observational associations and cannot determine whether persistent hypoperfusion is a reversible pathogenic process or a consequence of irreversibly injured tissue. Future trials should assess microcirculation at prespecified times after recanalization, distinguish incomplete large-vessel reperfusion from tissue no-reflow and hypoperfusion within established infarction, and test a mediation chain from improved tissue perfusion to reduced core growth and then better function. **Table 1** therefore classifies no-reflow as a measurable candidate phenotype with human observational support but without a targeted confirmatory RCT.

### 2.7 Regulated cell death: overlapping programs and a clinical evidence gap

#### 2.7.1 Apoptosis, necroptosis, and parthanatos

Regulated cell-death programs are not mutually exclusive and may vary with residual ATP, cell type, tissue compartment, and time after ischemia. Apoptosis is commonly associated with mitochondrial outer-membrane permeabilization, caspase activation, and DNA fragmentation and may occur in metabolically compromised tissue that is not immediately necrotic. Necroptosis is a lytic RIPK1-RIPK3-MLKL-associated program; necrostatin-1 reduced injury in a mouse model of ischemic brain injury (28). Parthanatos follows excessive PARP-1/2 activation after oxidative DNA damage, poly(ADP-ribose) accumulation, and apoptosis-inducing factor translocation; genetic studies support this pathway in experimental focal ischemia (29). Shared mitochondrial injury and release of danger signals make unambiguous attribution to one program difficult *in vivo* (29, 30).

#### 2.7.2 Ferroptosis and pyroptosis

Ferroptosis is an iron-dependent, lipid-peroxidative form of regulated cell death. In rodent models, tau-mediated iron export, brain iron accumulation, and lipid peroxidation have been linked to ischemic injury, while selenium-induced transcriptional adaptation including GPX4 improved experimental outcomes (31, 32). Pyroptosis is a gasdermin-mediated lytic program usually downstream of inflammasome and inflammatory-caspase activation; it couples membrane pore formation to release of IL-1β, IL-18, and other inflammatory signals (30). Ischemic tissue can display overlapping ferroptotic, pyroptotic, and nonspecific oxidative or inflammatory signatures, and neither pathway currently has a validated clinically specific biomarker in AIS.

#### 2.7.3 Scope and translational interpretation

The contemporary nomenclature of regulated cell death also includes mitochondrial permeability transition-driven necrosis, lysosome-dependent cell death, autophagy-dependent cell death, and additional context-specific programs (30). We emphasize apoptosis, necroptosis, ferroptosis, pyroptosis, and parthanatos because they have comparatively direct experimental links to cerebral ischemia-reperfusion and identifiable pathway components that could, in principle, support target-engagement assays. This selection is not intended to be exhaustive or to imply that any one program dominates human AIS. No acute-stroke RCT has yet shown that selective inhibition of one of these programs improves patient-centered outcomes.

**Table 1** synthesizes all major biological domains discussed in sections 2.1-2.7, including the broader regulated cell-death framework, their potential clinical roles, and the maturity of supporting evidence.

## 3 Reconstructing the translational paradigm of brain protection through lessons from classic failed trials

### 3.1 What do classic negative trials teach us?

#### 3.1.1 A single positive early study cannot replace independent confirmation

Over the past several decades, many interventions that appeared promising in animal models or early clinical studies subsequently failed in large trials. NXY-059 is a paradigmatic example. Despite a clear experimental rationale for free-radical trapping, SAINT II enrolled 3, 306 patients within 6 h of onset and did not improve either the 90-day mRS or NIHSS (33). This result does not show that oxidative stress is irrelevant to stroke; it shows that this particular combination of molecule, dose, exposure timing, and patient population failed to produce a clinical effect.

The ICTUS trial enrolled 2, 298 patients with moderate-to-severe AIS and found overall recovery with citicoline to be similar to placebo, leading the investigators to conclude that citicoline had no clinical efficacy under the conditions tested (34). High-dose albumin had been proposed to exert hemodynamic and cytoprotective effects in animal models, yet ALIAS Part 2 was stopped for futility after 841 patients; the primary outcome occurred in 44% of both groups, and pulmonary edema was substantially more common with albumin (35). These examples show that even when a candidate intervention has several plausible actions, inadequate clinical exposure, offsetting risk, or improved outcomes in the control group can reduce the net functional benefit to zero.

#### 3.1.2 Interactions with standard reperfusion therapy can alter the risk-benefit balance

A trial of recombinant human erythropoietin in 522 patients with AIS did not achieve its clinical objectives and showed increased mortality, raising particular safety concerns about combination with thrombolysis (36). The study demonstrates that an agent with putative tissue-protective properties can still produce net harm through prothrombotic effects, altered blood rheology, or interactions with standard therapy. URICO-ICTUS combined uric acid, an antioxidant, with alteplase and did not achieve a statistically significant difference in the primary excellent 90-day outcome (37). Labels such as “antioxidant, “ “anti-inflammatory, “ or “barrier protective” must therefore be judged by net clinical outcomes at a defined dose and within a defined standard-of-care context.

FAST-MAG challenged another simple assumption from the perspective of timing. Magnesium sulfate was initiated in the prehospital setting at a median of approximately 45 min, yet the 90-day mRS did not improve among 1, 700 patients with suspected stroke (38). The trial importantly established the feasibility of large-scale prehospital randomization, consent, and rapid drug delivery, but also showed that “earlier is better” addresses exposure timing only; it cannot compensate for an ineffective target, a mixed disease population, or insufficient pharmacologic intensity.

### 3.2 Why preclinical evidence tends to overestimate translational probability

#### 3.2.1 Mismatch between models, animals, experimental conditions, and patients

Early stroke studies often used young, healthy, male rodents with highly controlled occlusion and reperfusion times, whereas real-world patients are frequently older and have hypertension, diabetes, atrial fibrillation, prior brain injury, and multiple concomitant medications. The original STAIR recommendations called for dose-response testing, therapeutic-window assessment, histological and functional outcomes, and replication across laboratories and species before clinical translation (39). The 2009 update further emphasized randomization, blinded outcome assessment, sample-size estimation, prespecified exclusion criteria, and validation in female, aged, and comorbid animals (40).

ARRIVE 2.0 approaches the problem from the standpoint of reporting transparency, requiring clear descriptions of study design, sample size, inclusion and exclusion criteria, randomization, blinding, outcomes, and statistical analysis (41). Complete reporting does not guarantee an unbiased study, but missing information makes reproducibility and credibility difficult to judge. For candidate cytoprotective agents, preclinical development should also simulate modern co-treatment environments that include alteplase, tenecteplase, thrombectomy, anesthesia, and antithrombotic therapy.

Multicenter preclinical randomized controlled trials provide a further translational stress test. The PRISM Collective statement describes how coordinated studies across laboratories can incorporate biological and site heterogeneity, common data elements, harmonized core procedures, centralized or prespecified analyses, and prospective data-sharing plans while preserving justified local variation (42). By approximating selected features of phase III trials, mpRCTs can provide a more realistic estimate of robustness and between-site transportability before clinical investment, but they remain conditional on a valid mechanism, appropriate dose and exposure, and clinically meaningful assays.

#### 3.2.2 Reduced infarct volume is not equivalent to patient-centered benefit

Animal studies commonly use infarct volume at 24–72 h as a primary endpoint, whereas patients are most concerned with survival, independent living, cognition, and quality of life. Infarct volume correlates with function but is strongly influenced by lesion location, functional-network involvement, and baseline reserve; lesions of similar volume in the internal capsule and in a nondominant cortical region can produce very different disabilities. Preclinical programs that report histology alone may therefore select interventions that reduce acute cell death but are insufficient to alter long-term behavior or network reorganization. Future animal studies should evaluate long-term function, white-matter injury, cognition, and safety and should avoid selecting only the most favorable time point.

### 3.3 Pharmacokinetics, target engagement, and the “biological dose”

#### 3.3.1 Administered dose is not equivalent to brain exposure

The nominal dose of a cytoprotective drug matters only if the agent reaches the target tissue and modulates the intended pathway. Reduced blood flow in ischemic regions can limit delivery of intravenous drugs, while the BBB may be relatively intact early and increasingly permeable later, producing stage-dependent differences in brain exposure and toxicity. Reperfusion can restore drug delivery, but may also occur after part of the target tissue has already become irreversibly injured. Clinical trials should therefore record plasma concentrations and the interval from drug administration to reperfusion and, whenever possible, establish pharmacodynamic measures that are detectable in the brain or periphery.

#### 3.3.2 The nerinetide–alteplase interaction as a methodological case study

The signal observed in the non-alteplase subgroup of ESCAPE-NA1 prompted re-evaluation of drug-drug interactions. Based on pharmacokinetic data from rats, nonhuman primates, and humans, a cross-species study suggested that nerinetide may undergo plasmin-related degradation after alteplase; altering the administration sequence or using a proteolysis-resistant analog preserved protection in animal models (18). This evidence supports a plausible pharmacologic hypothesis, but ESCAPE-NEXT remained negative even after excluding patients who had received prior thrombolysis (43). Together, these findings show that pharmacokinetic mechanisms can explain part of an observed result, but they cannot be used *post hoc* to “rescue” a negative trial without prospective clinical validation.

### 3.4 Trial design itself can amplify or obscure treatment effects

#### 3.4.1 Population heterogeneity and ceiling/floor effects

Patients with mild stroke often have a high rate of spontaneous favorable outcomes, leaving little absolute room for improvement by a candidate treatment. Conversely, patients with severe large-core infarction may have little salvageable tissue left when treatment begins. Combining these groups within a single population can dilute effects in opposite directions. Treatment context also matters: patients without reperfusion, patients treated mainly with IVT, and patients achieving successful thrombectomy have different pathophysiological trajectories and different opportunities for additional benefit. Future trials should prespecify stratification according to vascular status, core volume, collateral circulation, and recanalization outcome rather than defining the study population solely by NIHSS score and time from onset.

#### 3.4.2 Choice of primary endpoint changes the question a trial answers

An mRS score of 0–1 captures excellent recovery, mRS 0–2 emphasizes functional independence, full-shift ordinal analysis uses all disability categories, and sliding dichotomy defines different success thresholds according to baseline severity. Each approach can be appropriate, but their “positive rates” are not directly comparable. Imaging-based infarct volume and 72-h NIHSS are useful for mechanistic screening but cannot substitute for a 90-day patient-centered outcome. The IRIS trial of tocilizumab used infarct-volume growth at 72 h as the primary endpoint and can therefore address whether interleukin-6 (IL-6) receptor inhibition influences tissue progression, but this result alone cannot establish long-term clinical efficacy (44).

#### 3.4.3 Early termination, small samples, and multiple analyses

Early termination must be interpreted according to what it changes statistically. CHARM randomized 535 participants, but its primary efficacy analysis included 431 patients aged 18–70 years and the trial ended early because of slow recruitment (15). Early termination reduced precision and limited the ability to exclude a modest benefit or harm; it does not explain away the neutral point estimate or provide evidence of efficacy. Conversely, small exploratory studies with many imaging, biomarker, and functional analyses are vulnerable to chance-positive findings. Interpretive certainty should therefore match developmental stage, achieved sample size, and the prespecified outcome hierarchy.

## 4 Pharmacologic brain cytoprotection: from target validation to functional outcomes

### 4.1 PSD-95 inhibition: the complete evidence chain of the nerinetide program

#### 4.1.1 ENACT as human proof of concept for target modulation

ENACT administered NA-1 prophylactically to patients undergoing endovascular repair of cerebral aneurysms. Because the timing of embolic microischemic lesions was predictable, the study detected favorable signals in the number or volume of DWI lesions (17). The result established that PSD-95 inhibition could be tested in a human ischemic setting, but the iatrogenic nature of the lesions, prophylactic dosing, and relatively mild clinical deficits limit external validity for spontaneous large-vessel occlusion.

#### 4.1.2 ESCAPE-NA1: an overall negative result and the interaction hypothesis

ESCAPE-NA1 enrolled 1, 105 patients with anterior-circulation large-vessel occlusion planned for EVT. The proportions achieving mRS 0–2 at 90 days were 61.4% with nerinetide and 59.2% with placebo (adjusted RR 1.04), so the primary endpoint was negative (45). A favorable non-alteplase subgroup signal generated a prospectively testable interaction hypothesis (18), but it cannot redefine an overall negative trial as positive.

#### 4.1.3 ESCAPE-NEXT: prospective testing in patients without prior thrombolysis

ESCAPE-NEXT randomized 850 patients who had not received prior IVT and were planned for EVT. Approximately 45% of the nerinetide group and 46% of the placebo group achieved mRS 0–2, corresponding to an odds ratio (OR) of 0.97 (43). This prospective trial failed to replicate the non-thrombolysis subgroup signal from ESCAPE-NA1 and substantially reduced the plausibility that alteplase interaction was the sole explanation for the earlier failure. It also illustrates why a dedicated trial of a subgroup hypothesis is a necessary form of scientific self-correction rather than simple repetition.

#### 4.1.4 FRONTIER and individual-patient-data analyses of the early window

FRONTIER enrolled patients with suspected severe stroke in the prehospital setting within 3 h. A total of 532 participants received study drug; the modified intention-to-treat population included AIS, intracerebral hemorrhage, transient ischemic attack, and stroke mimics. The primary functional outcome occurred in 57% versus 58%, with no significant difference (46). A signal was observed in the subgroup with confirmed AIS who underwent reperfusion; a subsequent *post hoc* individual-patient-data meta-analysis of 690 participants across three nerinetide trials who met both an early-window criterion of 3 h and a reperfusion criterion yielded an adjusted OR of 1.48 (47). These findings support the hypothesis that very early treatment in tissue destined for reperfusion may be a more favorable setting for PSD-95 inhibition, but they still require a new confirmatory trial with prespecified eligibility criteria and a prespecified primary endpoint.

Taken together, ENACT, ESCAPE-NA1, ESCAPE-NEXT, FRONTIER, and the subsequent individual-patient-data meta-analysis show that the main value of the nerinetide evidence chain lies not only in the drug itself, but also in illustrating how disease context, administration sequence, pre-reperfusion exposure, and analytical hierarchy can alter trial results (17, 18, 43, 45–47). Nerinetide cannot currently be considered proven effective, but the PSD-95 target also cannot be dismissed simply on the basis of these results.

### 4.2 Nelonemdaz: dual pharmacology without phase III benefit

#### 4.2.1 Exploratory trends in SONIC

Nelonemdaz combines NMDA receptor modulation with free-radical scavenging. In the phase II SONIC trial, 209 EVT-treated patients were randomized and 183 completed primary-outcome assessment. The proportions with mRS 0–2 at 12 weeks were 54.1%, 61.5%, and 63.2% in the placebo, low-dose, and high-dose groups, respectively, but the overall difference was not significant (48). The 90% confidence intervals for both active-dose groups crossed the null value, so the study provided safety and dose-exploration signals rather than evidence of efficacy.

#### 4.2.2 Confirmatory results from RODIN

RODIN randomized 496 patients undergoing mechanical reperfusion at 24 centers in South Korea. The 12-week ordinal mRS analysis yielded a cOR of 0.95, and infarct volume also showed no favorable difference (49). The result did not reproduce the numerical trends from SONIC, emphasizing that having two theoretically attractive pharmacologic actions does not necessarily create a clinically meaningful multitarget effect. Timing of treatment initiation, pre-reperfusion exposure, and tissue state may still influence outcomes, but nelonemdaz should not be regarded as an effective adjunct to EVT unless new prospective evidence emerges.

### 4.3 Multitarget small molecules: recent positive trials and questions of external validity

#### 4.3.1 Butylphthalide and BAST

Butylphthalide has been proposed to affect mitochondrial function, oxidative stress, the microcirculation, and inflammation. BAST enrolled 1, 216 patients at 59 centers in China who were treated within 6 h and received IVT and/or EVT. Using baseline NIHSS-dependent mRS success thresholds, the 90-day favorable-outcome rate was 56.7% with butylphthalide versus 44.0% with placebo (OR 1.70), without a significant increase in serious adverse events (50). This represents one of the stronger positive clinical signals among contemporary multitarget agents.

Several points remain important when interpreting BAST. The primary endpoint was modified early after trial initiation; most participants underwent IVT and had relatively mild stroke, whereas EVT or bridging therapy accounted for only about one third; and all centers were located within a single country. The trial supports improvement of its prespecified outcome in the studied population, but it does not prove that mitochondrial or microcirculatory mechanisms mediated benefit and cannot automatically be generalized to all large-core strokes, late-window presentations, or different stroke systems.

#### 4.3.2 The edaravone dexborneol clinical program

TASTE randomized 1, 165 patients within 48 h of onset to edaravone dexborneol or edaravone alone. The proportions achieving mRS ≤1 at 90 days were 67.18% and 58.97%, respectively (OR 1.42) (51). Because the comparator was active treatment, the trial estimates the comparative effect of the combination versus edaravone, not the net effect versus inactive treatment. TASTE-SL evaluated a sublingual formulation in 914 patients treated within 48 h who did not undergo EVT; mRS ≤1 was achieved in 64.4% versus 54.7%, for a risk difference of 9.70 percentage points (52). The publication subsequently issued corrections to **Figures 2**, **3**, but the primary outcome was not withdrawn; the corrected version should therefore be cited.

**Figure 3:**
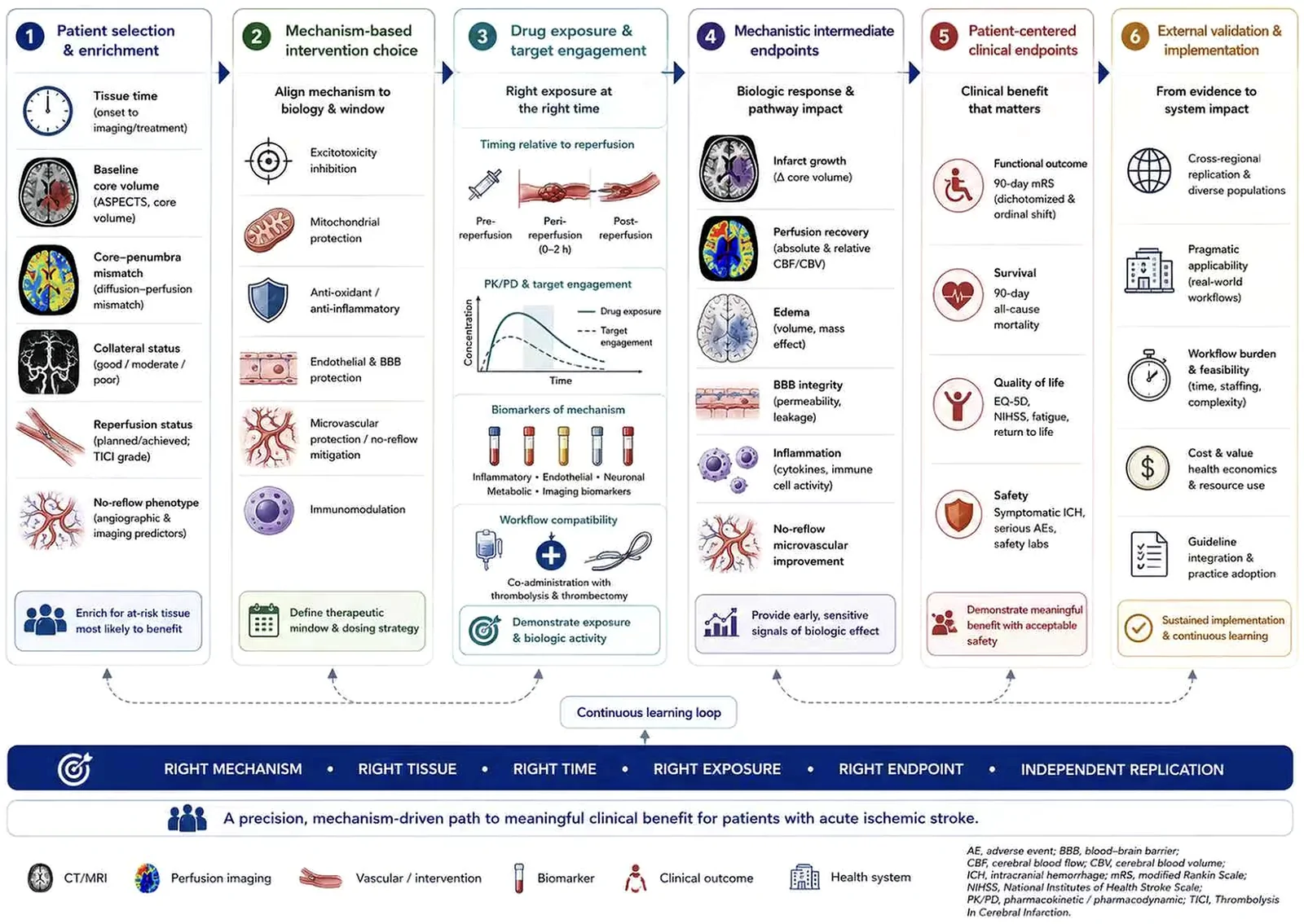
Translational roadmap for next-generation brain cytoprotection trials in the reperfusion era. A precision cytoprotection strategy should begin with enrichment for patients who retain an actionable tissue or pathophysiological phenotype, followed by selection of an intervention that matches the relevant mechanism and therapeutic window. Adequate exposure and target engagement should be demonstrated before progression to mechanistic intermediate endpoints, including infarct growth, tissue perfusion, edema, BBB integrity, inflammatory activity, and post-recanalization microcirculatory dysfunction. These signals should ultimately be linked to patient-centered functional outcomes and safety and then subjected to independent cross-regional validation and implementation assessment. The framework emphasizes the right mechanism, right tissue, right time, right exposure, right endpoint, and independent replication.

TASTE-2 extended the program into the EVT setting. Across 106 hospitals in China, 1, 362 patients with anterior-circulation large-vessel occlusion were randomized, with treatment initiated before thrombectomy. At 90 days, mRS 0–2 was achieved in 55.0% versus 49.6% (RR 1.11; risk difference 5.4 percentage points) (53). Statistical significance was borderline and the absolute effect was modest. A stronger signal and interaction were observed in the imaging-mismatch subgroup, but this remains a hypothesis of effect modification rather than a direct treatment indication. The consistent direction of three trials increases interest in this drug family, but differences in comparators, formulations, populations, and treatment windows prevent simple pooling.

#### 4.3.3 Minocycline and a wider treatment window

EMPHASIS enrolled 1, 724 patients with NIHSS scores of 4–25 within 72 h of onset at 58 hospitals in China. At 90 days, mRS 0–1 was achieved in 52.6% of the minocycline group and 47.4% of the placebo group (adjusted RR 1.11), and the full-shift mRS analysis was also favorable (54). This phase III result supports a clinical signal for a short course of minocycline in the studied population, but it does not prove that benefit was mediated by microglial inhibition, matrix metalloproteinase modulation, or BBB protection. The 72-h window also suggests a possible effect on secondary injury rather than exclusively on hyperacute penumbral preservation. Cross-regional replication, evaluation across different stroke severities, and mechanistic biomarker studies are therefore key next steps.

### 4.4 Strategies targeting inflammation, barrier injury, and cerebral edema

#### 4.4.1 ApTOLL: a comparatively direct path from target to clinic

TLR4-binding DNA aptamers showed receptor antagonism and protective effects *in vitro* and in murine ischemia models (21). The phase Ib/IIa ApTOLL trial compared two doses with placebo in 139 patients with anterior-circulation large-vessel occlusion treated within 6 h and scheduled for EVT. The 0.2 mg/kg dose had an acceptable safety profile and showed favorable directions across secondary outcomes including mortality, 72-h NIHSS, infarct volume, and 90-day mRS (22). Because the trial was small and designed primarily for safety and dose exploration, these findings constitute proof of concept rather than confirmatory efficacy.

#### 4.4.2 Counterevidence from targeting leukocyte trafficking

Natalizumab blocks α4-integrin-mediated leukocyte trafficking and therefore provides a relatively direct tool for testing whether restricting entry of peripheral immune cells into the brain improves stroke outcome. ACTION, with treatment within 9 h, did not reduce infarct growth, although exploratory functional signals were reported (24). ACTION II extended the treatment window to 24 h and compared 300 mg and 600 mg with placebo; it showed no functional benefit, and the probability of excellent or good outcomes was actually lower than with placebo (25). These findings suggest that broad inhibition of leukocyte trafficking may fail to target the dominant injurious process or may simultaneously suppress immune functions that are protective or reparative.

#### 4.4.3 IL-1 blockade, lymphocyte redistribution, and barrier protection

SCIL-STROKE administered a subcutaneous IL-1 receptor antagonist within 5 h. The trial primarily demonstrated safety and reduction of peripheral inflammatory markers but did not establish a 90-day functional benefit (23). Two small studies of fingolimod combined with alteplase reported lesion-, perfusion-, or function-related signals in patients treated within 4.5 h or during the 4.5–6 h window (26, 27). With only 47 and 46 participants, respectively, and limitations including open-label treatment, multiple endpoints, and single-region recruitment, these studies do not support routine immunomodulatory therapy.

3K3A-APC was designed to retain the cytoprotective and barrier-protective actions of activated protein C while reducing its anticoagulant activity. RHAPSODY used a randomized, blinded, dose-escalation design, established a maximum tolerated dose, and observed a trend toward less hemorrhage in exploratory analyses (55). The study primarily supports safety and further development rather than functional efficacy. It also illustrates a distinct translational pathway for biologics with clear mechanisms but potentially important safety risks: first establish tolerability in combination with thrombolysis and thrombectomy, and only then proceed to trials powered for functional effects.

#### 4.4.4 Emerging evidence for large-hemispheric edema and IL-6 receptor inhibition

CHARM evaluated intravenous glibenclamide in patients with large hemispheric infarction, with treatment initiated within 10 h and continued for 72 h. The 90-day ordinal mRS distribution was not improved in the prespecified modified intention-to-treat population, and hypoglycemia occurred in 6% versus 2% (15). Current evidence therefore does not support routine use for functional benefit. The early stop reduced precision around the effect estimate but, as discussed in section 3.4.3, does not convert the neutral result into evidence of efficacy.

The 2026 phase II IRIS trial evaluated a single 240-mg dose of tocilizumab in 108 patients undergoing EVT. Growth of the infarct core at 72 h was smaller in the tocilizumab group, with an adjusted mean difference after cube-root transformation of −0.41 (95% CI, −0.79 to −0.03); symptomatic intracranial hemorrhage, death, and serious adverse events did not show a clear increase (44). The study primarily provides a mechanistic signal that IL-6 receptor inhibition may influence tissue progression, and the investigators explicitly noted the need for further study of long-term functional outcomes. A positive imaging primary endpoint should not be presented as established clinical efficacy.

### 4.5 Vascular pleiotropic and cyclic-nucleotide approaches

#### 4.5.1 Statins

Statins have endothelial, antithrombotic, antioxidant, and anti-inflammatory actions beyond low-density-lipoprotein lowering, providing a plausible rationale for early use after AIS. In ASSORT, however, 257 patients with AIS and dyslipidemia were randomized to statin initiation within 24 h or on day 7; the 90-day mRS distribution did not differ (56). This result does not challenge the established role of statins in indicated secondary prevention, but it does not support timing of statin initiation as an acute cytoprotective intervention.

#### 4.5.2 Nitric oxide donors and phosphodiesterase inhibition

Nitric oxide-cGMP signaling can influence vascular tone, platelet activity, endothelial function, and microcirculation. Transdermal glyceryl trinitrate lowered blood pressure but did not improve 90-day function in ENOS, and ultra-early prehospital administration did not improve the overall functional outcome in RIGHT-2 (57, 58). Phosphodiesterase inhibitors are mechanistically heterogeneous. Cilostazol, a PDE3 inhibitor with antiplatelet and endothelial effects, was noninferior to aspirin in the 458-patient CAIST trial initiated within 48 h, but an active-comparator antiplatelet result does not demonstrate acute cytoprotection (59). Future studies should select the relevant PDE isoform, demonstrate cGMP/cAMP or perfusion target engagement, and distinguish acute tissue protection from antithrombotic secondary prevention.

## 5 Nonpharmacologic brain cytoprotection: low-cost feasibility and heterogeneous evidence

### 5.1 Remote ischemic conditioning

#### 5.1.1 Different “biological doses” under the same intervention name

Remote ischemic conditioning (RIC) uses repeated brief cycles of limb ischemia and reperfusion to activate putative humoral, neural, or immune-mediated remote protective responses. It is noninvasive, inexpensive, and potentially deployable before hospital arrival, but cuff site, inflation pressure, number of cycles, daily frequency, and total treatment duration vary greatly across studies. A single short prehospital RIC session is not biologically equivalent to bilateral upper-limb RIC administered twice daily for 10–14 days, and their effects should not be pooled simply because both are labeled RIC.

#### 5.1.2 Positive results from RICAMIS and their limits of applicability

RICAMIS enrolled 1, 893 patients with moderate AIS within 48 h at 55 hospitals in China and used five cycles of bilateral upper-limb ischemia-reperfusion twice daily for 10–14 days. The 90-day rate of mRS 0–1 was 67.4% versus 62.0% (OR 1.27) (60). Importantly, patients who had received IVT or EVT were excluded, and the trial used an open-label design with blinded endpoint assessment. The result therefore supports this specific multiday regimen in a defined moderate AIS population without reperfusion therapy and should not be directly extrapolated to adjunctive use with thrombolysis or thrombectomy.

#### 5.1.3 RESIST and pooled evidence

RESIST used a 20-mmHg sham procedure in patients with suspected stroke in the prehospital setting and continued treatment after hospital admission. The target population included both AIS and intracerebral hemorrhage, and the 90-day mRS was not improved either in the overall target population or in the AIS subgroup (61). A meta-analysis of 22 randomized controlled trials involving 7, 657 participants likewise found no consistent improvement in mRS with RIC in settings of routine medical therapy, IVT, or mechanical thrombectomy; NIHSS or recurrence signals in non-reperfused patients were secondary outcomes (62). Differences between RICAMIS and RESIST may reflect population, treatment intensity, duration, and blinding rather than a simplistic “East-positive, West-negative” pattern. Future trials should first define the most biologically plausible regimen and then test it with adequately rigorous sham control.

### 5.2 Normobaric hyperoxia

#### 5.2.1 Conceptual role as a tissue bridge before reperfusion

Normobaric hyperoxia does not remove a thrombus. Its purpose is to increase dissolved oxygen in plasma while patients are awaiting and undergoing reperfusion, temporarily augmenting oxygen delivery to hypoperfused tissue. It is therefore best conceptualized as “bridging tissue protection” rather than indiscriminate prolonged oxygen therapy. Potential risks include excessive oxidative stress, pulmonary intolerance, and differential effects across perfusion phenotypes; oxygen concentration, flow rate, and duration must therefore be explicitly defined.

#### 5.2.2 From a single-center pilot to OPENS-2

A 2022 single-center randomized pilot study in 86 patients with anterior-circulation large-vessel occlusion undergoing EVT found that normobaric hyperoxia added to standard EVT was associated with smaller infarct volume and directional functional signals (63). OPENS-2 subsequently enrolled 282 patients within 6 h at 26 centers in China, using 100% oxygen at 10 L/min for 4 h, with low-flow sham oxygen as the control. The adjusted cOR for the 90-day ordinal mRS analysis was 1.65, without a clear increase in mortality or serious adverse events (64). The progression from a single-center imaging signal to a multicenter functional endpoint is encouraging, but the sample remains limited and all participants were Han Chinese; the evidence is not yet sufficient for routine use.

### 5.3 Therapeutic hypothermia and selective brain cooling

#### 5.3.1 Implementation barriers to systemic hypothermia

Hypothermia can simultaneously reduce metabolic demand and modulate excitotoxicity, oxidative stress, and inflammation, producing broad protection in animal models. Clinical implementation, however, is constrained by cooling speed, shivering, sedation, intubation, pneumonia, arrhythmia, and rewarming. ICTuS 2 tested the feasibility of endovascular cooling in patients treated with alteplase, but the trial stopped early; mRS 0–1 was achieved in approximately 33% versus 38%, pneumonia occurred in approximately 19% versus 10.5%, and no functional benefit was demonstrated (65).

The European phase III EuroHYP trial stopped after 98 patients because of slow recruitment and funding problems. Only about 31% of participants reached the prespecified target temperature; the primary outcome was neutral and implementation and infection problems were again evident (66). These trials suggest that delayed cooling, failure to achieve target temperature, and complications can offset any biological effect. A strong mechanism cannot be fairly tested when the technical workflow cannot be delivered reliably.

#### 5.3.2 Early signals from selective intra-arterial hypothermia

Selective brain cooling aims to reduce the temperature of ischemic brain tissue rapidly through local cooling during thrombectomy while minimizing systemic adverse effects. The multicenter Chinese ISOLATION pilot trial enrolled 100 patients; mRS 0–2 at 90 days was achieved in 58% with selective intra-arterial hypothermia plus EVT and 40% with EVT alone (P = 0.055), which did not meet the conventional threshold for statistical significance (67). The study supports feasibility and a larger trial, but it does not establish efficacy.

A meta-analysis of 10 clinical studies involving 785 patients reported a pooled RR of 1.28 for mRS ≤2, with the lower confidence bound close to 1 and no reduction in mortality (68). Subgroups involving longer hypothermia or randomized studies showed increased risks of pneumonia and other complications. Because systemic and selective hypothermia as well as randomized and nonrandomized designs were pooled together, certainty is limited. Future trials should treat the cooling method, proportion achieving target temperature, rewarming strategy, and infection management as components of the intervention rather than reducing “hypothermia” to a binary variable.

*Representative clinical trials and the conclusions that can be supported by their results are summarized in* **Table 2**.

**Table 2:** Representative clinical trials of brain cytoprotection and the conclusions supported by their results.

Intervention/trial	n	Setting and phase	Main result	Evidence interpretation	Reference
NXY-059/SAINT II	3306	AIS within 6 h; phase III	mRS and NIHSS both negative	No clinical benefit for this specific free-radical trapping agent; does not invalidate oxidative stress as a target	(33)
Magnesium sulfate/FAST-MAG	1700	Suspected stroke within 2 h; prehospital	90-day mRS negative	Demonstrated feasibility of rapid prehospital trials; very early administration alone is insufficient	(38)
Nerinetide/ESCAPE-NA1	1105	EVT within 12 h	mRS 0–2: 61.4% vs 59.2%	Overall primary endpoint negative; non-thrombolysis subgroup is hypothesis-generating only	(45)
Nerinetide/ESCAPE-NEXT	850	EVT without prior IVT	mRS 0–2: 45% vs 46%	Did not replicate the non-thrombolysis subgroup signal	(43)
Nerinetide/FRONTIER	532	Suspected stroke within 3 h; prehospital	Primary mITT analysis negative	Signal in reperfused AIS subgroup requires prospective confirmation	(46, 47)
Nelonemdaz/SONIC	209	EVT within 8 h; phase II	No significant difference among three groups	Supports dose exploration and further study only	(48)
Nelonemdaz/RODIN	496	Mechanical reperfusion within 12 h; phase III	Ordinal mRS cOR 0.95	Did not improve function or infarct volume	(49)
Butylphthalide/BAST	1216	Within 6 h; IVT and/or EVT	Favorable outcome: 56.7% vs 44.0%	Positive in a defined Chinese population; mechanism and external validity require confirmation	(50)
Edaravone dexborneol/TASTE	1165	Within 48 h; active comparator	mRS ≤1: 67.18% vs 58.97%	Superior to edaravone alone; net effect versus placebo cannot be estimated	(51)
Sublingual edaravone dexborneol/TASTE-SL	914	Within 48 h; no EVT	mRS ≤1: 64.4% vs 54.7%	Positive in a defined non-EVT population; external replication is needed	(52)
Edaravone dexborneol/TASTE-2	1362	Within 24 h; dosing before EVT	mRS 0–2: 55.0% vs 49.6%	Modest, borderline overall benefit; mismatch subgroup remains exploratory	(53)
Minocycline/EMPHASIS	1724	AIS within 72 h	mRS 0–1: 52.6% vs 47.4%	Positive in a defined population; inflammatory mediation not demonstrated	(54)
ApTOLL	139	EVT within 6 h; phase Ib/IIa	Acceptable safety; several favorable secondary signals	Proof of concept, not confirmatory efficacy	(22)
Intravenous glibenclamide/CHARM	535	Large hemispheric infarction within 10 h	Ordinal mRS cOR 1.17; negative	Terminated early and more hypoglycemia; does not support routine functional benefit	(15)
Tocilizumab/IRIS	108	EVT within 24 h; phase II	Less infarct growth at 72 h	Imaging/mechanistic signal; long-term functional efficacy not established	(44)
RIC/RICAMIS	1893	Within 48 h; IVT/EVT excluded	mRS 0–1: 67.4% vs 62.0%	Positive for a specific multidose regimen; open-label and not generalizable to reperfusion adjunct use	(60)
RIC/RESIST	1500	Suspected stroke; prehospital; sham controlled	Target population and AIS subgroup negative	No benefit for the short-course/mixed-population regimen	(61)
Normobaric hyperoxia/OPENS-2	282	EVT within 6 h	Ordinal mRS cOR 1.65	Promising, but sample size and geographic scope are limited	(64)
Endovascular hypothermia/ICTuS 2	120	Background alteplase treatment	No improvement in mRS 0–1; more pneumonia	Feasible but no demonstrated functional benefit	(65)
Selective intra-arterial hypothermia/ISOLATION	100	Pilot adjunct to EVT	mRS 0–2: 58% vs 40%; P = 0.055	Feasibility and directional signal; underpowered for efficacy	(67)
Early statin therapy/ASSORT	257	AIS with dyslipidemia; within 24 h vs day 7	90-day ordinal mRS not improved	Pleiotropic rationale not confirmed as acute cytoprotection; secondary-prevention indications remain separate	(56)
Glyceryl trinitrate/ENOS and RIGHT-2	4011; 1149	Hospital treatment within 48 h; ultra-acute prehospital treatment	Blood pressure lowered in ENOS; overall functional outcomes neutral	NO-donor rationale did not translate into overall clinical benefit in these regimens	(57, 58)
Cilostazol/CAIST	458	AIS within 48 h; active aspirin comparator	Noninferior to aspirin for mRS 0-2	Supports an antiplatelet option, not proof of PDE3-mediated acute cytoprotection	(59)

## 6 Evidence integration in the reperfusion era: what forms of brain cytoprotection are more likely to succeed?

### 6.1 Brain cytoprotection does not represent a single clinical role

#### 6.1.1 Bridging cytoprotection before reperfusion

The first clinical role is to slow infarct-core growth while the occluded artery remains closed, thereby preserving tissue time for thrombolysis or thrombectomy. Such interventions must be initiated in the prehospital setting or very soon after imaging, and their effect is likely to depend heavily on the amount of residual penumbra. FRONTIER, FAST-MAG, and studies of normobaric hyperoxia represent different implementations of this concept (38, 46, 63, 64). FAST-MAG and FRONTIER demonstrate that earlier treatment alone is not sufficient; the sequential trials of normobaric hyperoxia suggest that tissue and functional signals may be easier to detect when an intervention directly targets hypoxic tissue and is tightly integrated with the EVT workflow.

#### 6.1.2 Modulation of peri-reperfusion injury

The second role is to reduce oxidative, inflammatory, barrier, and microcirculatory injury within a short window around recanalization. Nerinetide, ApTOLL, TASTE-2, tocilizumab, and selective intra-arterial hypothermia have all been designed to fit within the thrombectomy workflow (22, 43, 44, 53, 67). The advantages of this approach are that reperfusion timing can be documented, drug delivery to target tissue is more plausible, and core growth, barrier integrity, or perfusion can be used as mechanistic readouts. The challenge is that reperfusion itself has a large treatment effect, so the additional absolute benefit of adjunctive cytoprotection may be modest and may require more precise patient enrichment and larger sample sizes.

#### 6.1.3 Modulation of later secondary injury

The third role is to modulate persistent inflammation, edema, or cell death during the first 24–72 h. Minocycline, some edaravone dexborneol trials, and glibenclamide fall within this broader category (15, 51, 52, 54). These interventions may no longer primarily preserve the classical penumbra; instead, they may reduce secondary tissue loss or improve the biological environment for functional recovery. Pooling them with interventions delivered within 0–3 h under a single “neuroprotection” category would obscure fundamental differences in therapeutic targets and biological windows.

### 6.2 Mechanism, timing, and drug exposure must be aligned

#### 6.2.1 Clock time is only the crudest stratification variable

Time from onset to treatment remains important, but the rate of infarct-core growth varies markedly across patients. DAWN, DEFUSE 3, and penumbral imaging studies have shown that tissue status can diverge substantially from clock time (2, 3, 13, 14). Cytoprotection trials should therefore report baseline core volume, perfusion mismatch, collateral status, and final reperfusion rather than characterizing the population only as being treated “within 6 h” or “within 24 h.” Findings from early-window individual-patient-data analyses or imaging subgroups should be tested using new prospectively defined eligibility criteria rather than being converted directly into treatment rules after a negative parent trial.

#### 6.2.2 The drug-to-reperfusion interval may matter more than the drug-to-randomization interval

For agents that must reach the penumbra before reperfusion, rapid infusion after randomization does not necessarily provide adequate pre-reperfusion tissue exposure. Adjunct-to-thrombectomy trials should record study-drug initiation, arterial puncture, first effective reperfusion, and final reperfusion and prespecify exposure-by-time analyses. The nerinetide, TASTE-2, and ApTOLL programs illustrate that dosing sequence and workflow compatibility are components of the treatment strategy, not merely operational details (22, 43, 53).

#### 6.2.3 Target engagement should connect imaging with functional outcomes

An ideal translational chain comprises effective exposure, modification of a prespecified mechanistic biomarker, attenuation of tissue injury, and ultimately improved patient function. Most existing trials cover only one or two of these links. SCIL-STROKE demonstrated changes in peripheral inflammation without establishing functional benefit (23); IRIS showed reduced infarct growth while long-term functional efficacy remains to be established (44); and ApTOLL produced directionally favorable imaging and functional signals but was underpowered for confirmatory efficacy (22). Future studies should prespecify mediation analyses while avoiding the interpretation of mediator associations as proof of causality.

### 6.3 Patient selection should shift from “disease labels” to “actionable phenotypes”

#### 6.3.1 Vascular and tissue phenotypes

AIS is a highly heterogeneous syndrome in etiology, lesion location, severity, and opportunity for reperfusion. Patients with anterior-circulation large-vessel occlusion, distal-vessel occlusion, lacunar infarction, or no identifiable occlusion should not be assumed to have the same cytoprotective needs. For adjuncts to thrombectomy, baseline core volume, mismatch, collateral status, reperfusion grade, and postprocedural perfusion may provide more direct biological stratification than the NIHSS alone. For patients who do not undergo reperfusion therapy, persistent hypoperfusion, progressive symptoms, or inflammatory/edematous phenotypes should be explicitly characterized.

#### 6.3.2 No-reflow as a candidate enrichment phenotype

Two recent studies indicate that tissue-level hypoperfusion is not uncommon after technically successful thrombectomy and is associated with outcomes (6, 7). However, thresholds and imaging time points vary across studies, precluding a uniform enrichment strategy at present. The next step is to establish a reproducible multicenter definition, determine whether early postprocedural no-reflow is reversible, and use improved tissue perfusion as a mechanistic endpoint, core growth as an intermediate outcome, and 90-day mRS as the clinical endpoint. Without this hierarchical chain, no-reflow risks becoming an overly broad label used to explain any unfavorable outcome.

#### 6.3.3 Biomarkers must combine speed with specificity

Acute stroke care cannot accommodate time-consuming, complex omics screening; clinically useful biomarkers must therefore be obtainable without delaying reperfusion. Potential candidates include rapid blood-based inflammatory or endothelial markers, drug concentrations, perfusion and barrier imaging, and postprocedural core growth. Their value lies not merely in prognostic association but in predicting treatment effect or confirming target engagement. Observing that a biomarker is elevated in patients with poor outcomes does not establish that lowering that biomarker will improve outcome.

### 6.4 Primary endpoints and statistical interpretation determine evidential strength

#### 6.4.1 Prespecified primary endpoints take precedence over *post hoc* findings

The overall negative result of ESCAPE-NA1 cannot be overturned by the non-thrombolysis subgroup, the negative mITT analysis of FRONTIER cannot be replaced by its reperfused subgroup, and the modest overall effect in TASTE-2 cannot be redefined as a large effect on the basis of the mismatch subgroup (45, 46, 53). The appropriate purpose of subgroup analyses is to generate testable hypotheses. Their credibility depends on whether they were prespecified, whether a formal interaction is present, whether the direction of effect is mechanistically coherent, and whether the finding can be independently replicated.

#### 6.4.2 mRS, imaging, and short-term neurological outcomes have distinct limitations

Ordinal analysis across the full mRS range is appropriate for assessing shifts across the entire disability spectrum, whereas dichotomies such as mRS 0–1 or 0–2 are clinically intuitive but may be affected by ceiling or floor effects. Infarct volume and core growth can increase the efficiency of phase II trials but depend on lesion location and imaging methodology. The NIHSS is sensitive to language and motor deficits and to anterior-circulation large-vessel stroke, but less comprehensively captures cognition, fatigue, and posterior-circulation symptoms. Future cytoprotection trials should define a patient-centered primary endpoint and position imaging and mechanistic readouts as hierarchically organized secondary endpoints rather than selecting the most favorable secondary result after a negative primary endpoint.

#### 6.4.3 Absolute effect size and implementability are equally important

When standard reperfusion therapy already produces a large benefit, the incremental effect of cytoprotection may be only a few percentage points. Even a statistically significant result must therefore be interpreted alongside treatment cost, administration complexity, adverse events, and any delay imposed on the stroke workflow. The approximately 5.4-percentage-point risk difference in TASTE-2, the hypoglycemia risk in CHARM, and the pneumonia and procedural burden associated with hypothermia all illustrate why effect size and implementation cost must be considered together (15, 53, 65, 66).

### 6.5 External validity, independent replication, and guideline adoption

#### 6.5.1 Internal validity of single-country multicenter trials and their external boundaries

Multicenter randomization can strengthen internal validity but cannot fully eliminate geographic concentration in healthcare systems, population composition, standard treatment, and rehabilitation resources. Trials of butylphthalide, edaravone dexborneol, minocycline, RICAMIS, and OPENS-2 provide important evidence, but the generalizability of their findings requires evaluation in different countries and stroke systems (50–54, 60, 64). Limited external validity does not imply that the results are unreliable; rather, it means that an additional step remains between “effective in this trial” and “routinely effective worldwide”.

#### 6.5.2 Negative replication is not wasteful but essential to evidence maturation

The failure of ESCAPE-NEXT to confirm the ESCAPE-NA1 subgroup hypothesis, of RODIN to confirm the SONIC numerical trend, and of ACTION II to reproduce the early signals from ACTION all strengthened the field’s evidential judgment (24, 25, 43, 48, 49). Conversely, when successive positive trials arise from the same drug-development program, similar populations, and the same region without independent-team validation, reproducibility and implementation context remain legitimate concerns. Guideline change requires more than a P value; it also requires stability of effect, safety, generalizability, and compatibility with standard care pathways.

*The principal translational barriers and corresponding recommendations for next-generation study design are summarized in* **Table 3**.

**Table 3:** Major translational barriers and recommendations for next-generation study design.

Translational barrier	Common manifestation	Suggested research strategy	Representative evidence
Overly idealized preclinical models	Young healthy animals, fixed occlusion times, lack of co-treatment	Multicenter preclinical randomized controlled trials; include female, aged, hypertensive, and diabetic models; simulate IVT/EVT; prespecify intersite analyses and data sharing	(39–42)
No salvageable tissue at the time of treatment	Enrollment based only on clock time	Prespecified enrichment by baseline core, mismatch, collateral status, and core-growth rate	(2, 3, 13, 14)
Uncertain brain exposure and target engagement	Only administered dose and clinical outcome reported	Pharmacokinetic sampling, drug-to-reperfusion timing, rapid pharmacodynamic markers, and mediation analysis	(10, 18, 23, 44)
Interaction with standard treatment	Thrombolysis, thrombectomy, anesthesia, and antithrombotic therapy omitted from pharmacologic design	Test administration sequence and compatibility with co-treatment in preclinical and early clinical phases	(18, 36, 43, 45)
Heterogeneous populations and pathological phenotypes	Treating all AIS as a single population	Prespecified stratification by vascular status, reperfusion, no-reflow, edema, or inflammatory phenotype	(6, 7, 50, 53, 60)
Disconnect between surrogate endpoints and function	Positive infarct-volume or short-term NIHSS result treated as proof of clinical efficacy	Patient-centered primary endpoint; imaging and biomarkers as hierarchical mechanistic endpoints	(22, 23, 44, 49)
Small samples, multiple analyses, and early termination	Exploratory signals are overinterpreted	Control multiplicity; prespecify interactions; report stopping reasons and conditional power; obtain independent confirmation	(15, 47, 53, 67)
Geographic concentration and implementation differences	Positive single-country results generalized directly	Cross-regional independent replication; evaluate workflow delays, cost, and real-world implementability	(4, 50–54, 64, 69)

Integrating these considerations, **Figure 3** proposes a mechanism-driven translational roadmap that links actionable patient phenotypes, therapeutic timing, drug exposure and target engagement, mechanistic intermediate endpoints, patient-centered outcomes, and independent external validation.

## 7 Discussion: controversies, limitations, and future research directions

### 7.1 Controversies that most urgently require clarification

#### 7.1.1 Are multitarget strategies superior to single-target strategies?

Several recent trials of multitarget small molecules have reported positive results, whereas multiple trials directed at excitotoxicity have been negative, creating an impression that multitarget approaches are necessarily superior to single-target approaches. Direct head-to-head evidence does not currently support that conclusion. Single-target strategies offer mechanistic clarity and easier verification of target engagement but may be insufficient to cover the networked nature of ischemic injury. Multitarget drugs may affect several pathways simultaneously, yet their active components, optimal dose, and mediators of benefit may be difficult to identify. Future comparisons should therefore focus not on labels but on whether a prespecified biological target can be demonstrated in patients and whether it yields reproducible functional benefit.

#### 7.1.2 Do hyperacute cytoprotection and delayed treatment belong to the same category?

Nerinetide or normobaric hyperoxia delivered within 0–3 h primarily aims to preserve the pre-reperfusion penumbra, whereas minocycline or edema-directed treatment initiated at 24–72 h is more likely to influence secondary injury. Combining these strategies into a single “neuroprotective effect” introduces substantial clinical heterogeneity. Future studies should first define the clinical role of the intervention and then determine the appropriate time window and endpoints; meta-analyses should likewise stratify by clinical role rather than pool interventions solely because they carry a neuroprotection label.

#### 7.1.3 Regionally positive trials versus a cautious international guideline position

Several multicenter trials from China provide high-quality randomized evidence and should not be discounted because of geography. Guideline adoption, however, also requires cross-regional replication, consideration of standard care in the control group, absolute effect size, and implementation feasibility. The 2026 AHA/ASA guideline still does not support neuroprotection as an undifferentiated routine treatment category (4), whereas several recent studies were published close to or after the evidence-integration window for that guideline (44, 53, 54, 69). A balanced approach is to update the evidence intervention by intervention rather than using a class-level guideline statement to dismiss all new data or using a single positive trial to declare that the field has achieved a definitive breakthrough.

### 7.2 Limitations of the current evidence base

#### 7.2.1 Mechanistic evidence remains predominantly preclinical

Evidence for NOX4, pericyte contraction, mitochondrial dysfunction and intercellular transfer, and the regulated cell-death programs discussed above derives mainly from cellular or animal studies (5, 19, 20, 28–32). These studies are valuable for mapping injury networks but cannot establish the dominant mechanism, cellular source, or clinical reversibility in patients. Human blood and imaging surrogates often lack cell specificity; accordingly, this review uses terms such as “suggests, “ “may contribute, “ and “candidate target” for these mechanisms.

#### 7.2.2 Heterogeneity across clinical trials remains difficult to eliminate

Trial populations range from mild-to-moderate non-reperfused stroke to large-core EVT populations; treatment initiation ranges from approximately 45 min in the prehospital setting to 72 h; and primary endpoints include mRS 0-1, mRS 0-2, sliding dichotomy, ordinal analysis, and infarct growth. Even when statistical pooling is possible, the resulting average effect may not correspond to an executable clinical strategy. A recent meta-analysis of adjunctive therapies in EVT reported an odds ratio of 1.13 for functional independence, but the authors explicitly cautioned against interpreting this small, heterogeneous signal as evidence that “neuroprotection as a class is effective” (69).

#### 7.2.3 Limitations of this review

This is a narrative review based on a predefined core evidence set supplemented by literature directly relevant to the central argument. We did not undertake preregistration, dual independent screening, RoB 2 assessment, or GRADE evaluation. Additional references were included to strengthen coverage of foundational concepts, discordant findings, and translational pathways rather than to claim exhaustive inclusion of all candidate therapies. Pediatric stroke, hemorrhagic stroke, chronic neural repair, cell-based therapy, and long-term rehabilitation are not reviewed systematically; the conclusions therefore apply specifically to early brain cytoprotection in adult AIS.

### 7.3 Five priorities for future research

#### 7.3.1 Establish a reproducible definition of no-reflow in humans

Postprocedural CT perfusion, MR perfusion, flat-panel CT, and other rapid modalities should be compared to define the optimal measurement time, thresholds, and relationship with final infarction. Mechanistic trials should then enrich for patients with demonstrable no-reflow and test whether an intervention first improves tissue perfusion, subsequently reduces core growth, and ultimately improves function. Two recent human studies provide a phenotypic foundation for this direction, but causal validation remains incomplete (6, 7).

#### 7.3.2 Incorporate target engagement into early clinical decision-making

Early-phase trials should ask not only whether an intervention is safe and whether the mRS shows a favorable trend, but also whether the drug achieves effective exposure and modifies the prespecified pathway. Infarct growth in the tocilizumab trial, inflammatory markers in SCIL-STROKE, and pharmacokinetic interactions in the nerinetide program illustrate imaging-, biomarker-, and pharmacokinetic-based approaches, respectively (18, 23, 44). Future programs could use explicit go/no-go criteria: failure to engage the target should preclude progression to a large phase III trial, whereas target engagement without tissue or functional change should prompt reassessment of the mechanism’s clinical importance.

#### 7.3.3 Develop multicenter preclinical studies and platform clinical trials

Preclinical programs should follow STAIR and ARRIVE principles and, for candidates approaching clinical translation, adopt multicenter preclinical randomized controlled trials (mpRCTs). The PRISM Collective recommends shared protocols and common data elements, prospective randomization and blinding, adequate sample size, explicit handling of intersite heterogeneity, inclusion of biologically diverse models, prespecified analyses, and transparent data sharing (39–42). mpRCTs can test robustness across laboratories and contexts, although they cannot compensate for an invalid target or an assay that does not measure target engagement. Clinical development can then use adaptive or platform trials with prespecified decision rules to compare mechanistically distinct candidates within a common imaging, timing, and outcome framework.

#### 7.3.4 Embed treatment into prehospital and thrombectomy workflows without causing delay

Interventions that require hyperacute exposure can be delivered through ambulances, mobile stroke units, or prehospital remote randomization, whereas adjuncts to thrombectomy should be initiated immediately after imaging without delaying arterial puncture. Trials should treat workflow metrics as safety outcomes, including door-to-needle, door-to-puncture, and reperfusion times. Any theoretically effective cytoprotective strategy that delays reperfusion may produce a negative net clinical effect.

#### 7.3.5 Conduct cross-regional independent replication and implementation evaluation

For a positive phase III trial conducted within a single country, the next step should be replication by independent investigators across different stroke systems, including evaluation of effect stability under different thrombolytic agents, anesthesia strategies, thrombectomy techniques, and rehabilitation resources. When the treatment effect is only a few percentage points, cost, administration complexity, adverse events, and the resources required to achieve one additional patient with functional independence should be evaluated concurrently. Moving brain cytoprotection from statistical significance to clinical value requires joint validation of efficacy, implementability, and equitable accessibility.

## 8 Conclusion

Reperfusion therapies have conclusively demonstrated that ischemic brain tissue can be salvaged and have provided brain cytoprotection research with a much clearer background of restored blood flow than existed in earlier eras. Nevertheless, current evidence does not support grouping interventions that differ in mechanism, therapeutic window, and clinical role into a universally effective “neuroprotection class.” Studies of NXY-059, citicoline, albumin, magnesium sulfate, nerinetide, and nelonemdaz show that preclinical plausibility, very early administration, or favorable signals in early trials are insufficient to guarantee functional benefit. Studies of natalizumab, IL-1 receptor antagonism, glibenclamide, and hypothermia further show that effects on inflammatory, imaging, or edema-related endpoints do not necessarily translate into independence at 90 days.

Conversely, trials of butylphthalide, edaravone dexborneol, minocycline, ApTOLL, tocilizumab, and normobaric hyperoxia suggest that selected interventions may produce clinical or mechanistic signals in specific patients, tissue states, and reperfusion settings. Their shared limitations are insufficient external replication, incomplete demonstration of mechanistic mediation, and limited precision in patient selection. The next phase of the field should not simply expand the list of unscreened candidate drugs; instead, it should build falsifiable precision-cytoprotection strategies around the “right mechanism, right tissue, right time, right exposure, right endpoint, and independent replication.” Only when this complete evidence chain is prospectively validated can brain cytoprotection move from a longstanding translational aspiration toward scalable stroke practice.

## Glossary

AIF: apoptosis-inducing factor
AIS: acute ischemic stroke
BBB: blood-brain barrier
cGMP: cyclic guanosine monophosphate
cOR: common odds ratio
CT: computed tomography
DAMP: danger-associated molecular pattern
DWI: diffusion-weighted imaging
EVT: endovascular thrombectomy
GSDMD: gasdermin D
GTN: glyceryl trinitrate
IL: interleukin
IVT: intravenous thrombolysis
mITT: modified intention-to-treat
MLKL: mixed-lineage kinase domain-like protein
mpRCT: multicenter preclinical randomized controlled trial
MRI: magnetic resonance imaging
mRS: modified Rankin Scale
NIHSS: National Institutes of Health Stroke Scale
NMDA: N-methyl-D-aspartate
NOX4: NADPH oxidase 4
OR: odds ratio
PARP: poly(ADP-ribose) polymerase
PDE: phosphodiesterase
PSD-95: postsynaptic density protein 95
RCT: randomized controlled trial
RIC: remote ischemic conditioning
RIPK: receptor-interacting protein kinase
RR: risk ratio
TLR4: Toll-like receptor 4.
